# Nanotechnology as a Processing and Packaging Tool to Improve Meat Quality and Safety

**DOI:** 10.3390/foods10112633

**Published:** 2021-10-29

**Authors:** Melisa Lamri, Tanima Bhattacharya, Fatma Boukid, Imene Chentir, Amira Leila Dib, Debashrita Das, Djamel Djenane, Mohammed Gagaoua

**Affiliations:** 1 Laboratory of Food Quality and Food Safety, Department of Food Technology, Université Mouloud Mammeri, Tizi-Ouzou 15000, Algeria; lamrimeliza1@gmail.com (M.L.); djenane6@yahoo.es (D.D.); 2 Innovation, Incubation & Industry (I-Cube) Laboratory, Techno India NJR Institute of Technology, Udaipur 313003, India; btanima1987@gmail.com; 3 Food Safety and Functionality Programme, Institute of Agriculture and Food Research and Technology (IRTA), 17121 Monells, Spain; fatma.boukid@irta.cat; 4 Laboratory of Food, Processing, Control and Agroressources Valorization, Higher School of Food Science and Agri-Food Industry, Algiers 16200, Algeria; i.chentir@essaia.dz; 5 GSPA Research Laboratory, Institut des Sciences Vétérinaires, Université Frères Mentouri, Constantine 1, Constantine 25000, Algeria; dibamira@hotmail.com; 6 School of Community Science & Technology, IIEST Shibpur, Howrah 711103, India; debashritadas95@gmail.com; 7 Food Quality and Sensory Science Department, Teagasc Food Research Centre, Ashtown, D15 KN3K Dublin, Ireland

**Keywords:** meat, food packaging, nanotechnology, silver nanoparticles, green technology, spoilage, food safety, nanosensors

## Abstract

Nanoparticles are gaining momentum as a smart tool towards a safer, more cost-effective and sustainable food chain. This study aimed to provide an overview of the potential uses, preparation, properties, and applications of nanoparticles to process and preserve fresh meat and processed meat products. Nanoparticles can be used to reinforce the packaging material resulting in the improvement of sensory, functional, and nutritional aspects of meat and processed meat products. Further, these particles can be used in smart packaging as biosensors to extend the shelf-life of fresh and processed meat products and also to monitor the final quality of these products during the storage period. Nanoparticles are included in product formulation as carriers of health-beneficial and/or functional ingredients. They showed great efficiency in encapsulating bioactive ingredients and preserving their properties to ensure their functionality (e.g., antioxidant and antimicrobial) in meat products. As a result, nanoparticles can efficiently contribute to ensuring product safety and quality whilst reducing wastage and costs. Nevertheless, a wider implementation of nanotechnology in meat industry is highly related to its economic value, consumers’ acceptance, and the regulatory framework. Being a novel technology, concerns over the toxicity of nanoparticles are still controversial and therefore efficient analytical tools are deemed crucial for the identification and quantification of nanocomponents in meat products. Thus, migration studies about nanoparticles from the packaging into meat and meat products are still a concern as it has implications for human health associated with their toxicity. Moreover, focused economic evaluations for implementing nanoparticles in meat packaging are crucial since the current literature is still scarce and targeted studies are needed before further industrial applications.

## 1. Introduction

The requirements of consumers for a high food quality along with their focus on health and wellness have triggered an exceptional development and innovation in food processing and packaging, including in the meat production sector [1]. Therefore, a large use of additives and ingredients endowed with nutritional and functional features were investigated to improve the sensory, visual, and aromatic quality properties and to avoid the spoilage of food products resulting in wastage reduction [2,3]. During food processing including that of meat products, additives like antioxidants, binders and thickeners, humectants, curing agents (sodium nitrite and nitrate), flavor enhancers, tenderizing enzymes are incorporated to improve the quality or to display functional qualities of the final products [4,5,6,7,8]. However, several of the ingredients used by food and meat industry to stabilize the quality and extend the shelf-life of the products can be associated with health issues, especially when consumers are exposed to high additive concentrations [9]. To these aspects, some of the conventional packaging used by the industry could represent a real health risk when the synthetic antioxidants migrate from the packaging to the packed food [10].

Based on the above, there is a continuous shift in the demand of modern consumers towards the development of novel meat and packed meat products with low amounts of synthetic additives along with increased use of natural bioactive components with positive health effects. Therefore, the incorporation of synthetic additives and bioactive components in their native states and shapes could show a low availability leading to a lower functionality and efficiency, hence making the meat and packed meat products of poor sensory quality with reduced shelf-life. This fact led to the use of an increased number of natural substances to ensure the availability of molecules and an enhancement of product quality with lower costs [11]. Therefore, there was an interest to search for safer and low-cost alternative solutions that can be used for meat products processing and packaging. In this sense, the meat industry needs to implement innovative alternatives to render these additives and bioactive components more available by changing their shape and size properties to improve the overall quality (sensory and safety) of fresh meat and packed meat products [4].

Nanotechnology emerged as an innovative alternative that is increasingly applied in the meat production chain to ensure a longer shelf-life with improved food sensory and safety quality and traceability [11,12]. It can be defined as a technology area aiming to elaborate nano-sized materials of less than 100 nm that possess unique and novel properties [1,4]. Due to their high surface area to volume ratio, nano-sized materials reach and act more efficiently on their target at very low concentrations [13].

Looking to the recent literature, it seemed that the incorporation and use of bioactive and functional nanomaterials in meat products has generated significant amount of research in the area of nanotechnology [14]. For example, the recent study by Sani et al. [15] prepared an active film packaging using potato starch and apple peel pectin to which the microencapsulation of essential oil from *Zataria multiflora* and zirconium oxide (ZrO_2_) nanoparticles allowed an efficient preservation of quail meat and positively increased the shelf-life of the product. Similarly, Priyadarshi et al. [16] prepared using the casting approach a carboxymethyl cellulose-based functional films to which they incorporated zinc oxide nanoparticles (ZnONPs: a very efficient and safe additive) and grape seed extracts and allowed an excellent antioxidant activity and suitability to protect high-fat beef samples. Indeed, ZnONPs not only improve the thermal, mechanical, and water vapor barrier properties of the base polymer that can be prepared using different methods, but also are known to have an excellent antibacterial activity against a wide range of food pathogens, hence allowing to extend the shelf-life of packaged meat products [16]. Moreover, ZnONPs are generally recognized as safe (GRAS) from the United States Food and Drug Administration, making it suitable for food contact applications [17]. Silver nanoparticles (AgNPs, referred as E174 in the food industry in the EU) are also widely used to improve the barrier, mechanical, and antibacterial properties of food packages, as well as to maintain the quality of food and meat products under the regulation of the United States Food and Drug Administration and the European Food Safety Authority [18].

Overall, the application of nanotechnology in meat processing and packaging was performed for several objectives: (i) protection of products against microbial spoilage, (ii) improvement of sensory properties, (iii) upgrade of functional and nutritional aspects of the meat products, and (iv) to monitor the quality during storage [19,20]. However, nanotechnology is still a controversial subject for the general public, who had more questions than answers, due to the lack of knowledge and regulations [21]. Thus, the worth of implementating nanotechnology in meat processing industries depends largely on the economic significance of nanotechnology, acceptance by the consumers, and the consideration of certain regulations pertaining to the application of this technology [21]. In this light, the present article intends (i) to summarize the current knowledge on the application of nanotechnology meat processing and packaging as well as (ii) to provide an overview about the pros and cons of materials used in the synthesis of nanoparticles, (iii) a summary of the main mechanisms and sources of the nanomaterials, (iv) the different approaches of nanoparticles applications in meat products processing and packaging, and (v) the currently available literature about the potential toxicity issues related to synthesized nanoparticles used for meat processing and packaging.

## 2. Types and Synthesis Techniques of Nanoparticles

**Figure 1:**
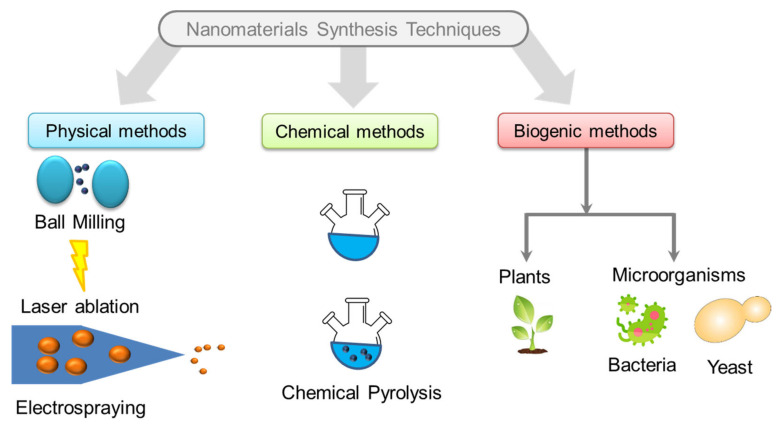
Schematic diagram of few common techniques of nanomaterial synthesis.

Nanoparticles (NPs) are a wide class of materials that includes particulate substances, which have a dimensions less than 100 nm at least. NPs are divided into various categories depending on their morphology, size, and chemical properties [13]. Thus, NPs can be classified in two main classes, namely inorganic nanoparticles and organic nanoparticles that can be shaped into nanotubes, nanofibers, nanoemulsions, etc., using organic materials such as biopolymers, oils, carbon, etc. [11]. Nanoparticles can be synthesized using several techniques including physical, chemical, and biogenic methods [22] (Figure 1) such as ball milling [23], electrospraying [24], microwave, spark discharge or laser ablation [25], condensation of inert gas, sol-gel, chemical and physical vapor deposition, and nanoemulsion [26]. Nanoparticles are also derived with the help of biological sources like bacteria, algae, fungi, etc. [27]. The selection of the synthesis technique depends on the desired type of nanoparticles to synthetize. Based on physico-chemical properties, the most well-known classes of NPs and the largest reported in meat processing and packaging are detailed in the following sections.

### 2.1. Inorganic Nanoparticles

Inorganic NPs, such as magnetic, quantum dots, ceramic, and metallic NPs, are characterized by a central core, which is composed by inorganic particles [18]. Inorganic NPs are endowed with interesting magnetic, optical, electronic, or fluorescent properties. The synthesis of inorganic NPs with magnetic and electronic particles must be performed in a tailored manner facilitating a proper control of the size and shape of the synthesized nanoparticles. The main methods of synthesis are precipitation of the salts in aqueous medium, hydrothermal synthesis, microemulsions, decomposition in organic media, polyol process, and aerosol pyrolysis [26,28,29]. The precipitation of salt in an aqueous medium is mostly used for producing magnetic nanoparticles. Polyol, decomposition in organic media, etc., are also commonly used for producing magnetic nanoparticles. Recently, microfluidic technology has been used for the purpose of synthesizing inorganic nanomaterials with narrower size compared to bulk methods [30]. On the other hand, the process of nebulizing on stirred liquid surface generated a solid phase of nanosized particles [31]. It is worth mentioning that it was reported that aerosol-assisted wet chemical methods are more efficient and less time-consuming than flame spray pyrolysis [32].

### 2.2. Organic Nanoparticles

Organic NPs are made of polymeric or lipid compounds. Compared to inorganic NPs, the organic ones received less attention. Organic NPs are eco-friendly, economical, and suitable for biological applications [33]. The organic NPs could be synthesized following the emulsification process, nanoprecipitation, and drying procedures. The emulsification process consists of solubilizing organic substances to form nanodroplets with a defined size and then forming nanoparticles using various techniques including polymerization, precipitation, etc. Moreover, synthesizing organic NPs was performed using spray drying, piezoelectrical technology, and supercritical fluid [34,35]. Synthetic chemistry enabled the fabrication of nanoparticles from molecules and self-organization to facilitate the formation of various systems (e.g., liposomes, micelles, capsules, polymeric nanoparticles). Organic nanoparticles have the ability of loading molecules through encapsulation (physically or by surface or core conjugation) indicating their potential use for specific molecule delivery. In this context, a study by Pabast et al. [36] designed a new coating structure for lamb meat by Satureja essential oil (SKEO)-loaded nanoliposomes. The authors reported that incorporating chitosan coating containing nano-encapsulated SKEO in lamb meat led to the retention of the high-quality properties, improvement of microbiological safety, and extension of shelf-life during chilled storage as well as a better oxidative stability. Similar conclusions were achieved in earlier studies, indicating that encapsulation of essential oils in liposomes produced more antimicrobial and antioxidant activity than their use in their native forms [37,38]. Therefore, this can explain the reduced evaporation properties and facility of delivery to the bacterial cell wall.

### 2.3. Biopolymeric Nanoparticles

Biopolymeric nanoparticles were first designed by using biopolymers such as albumin, and non-biodegradable synthetic polymers like polyacrylamide and polymethyl acrylate [39]. They have a particle size ranging from 1 to 1000 nm and can be loaded using different biopolymers [40]. The main objective of preparing biopolymer nanoparticles is to avoid toxicity of non-degradable polymers, which are threats to humans as well as the environment. Proteins, starch, and lipids are the major sources of these kinds of nanoparticles. Proteins like whey, zein, and soybean are fabricated as nanomaterials for various food packaging applications including muscle meats and meat products [15,41,42,43]. Chitosan, a complex carbohydrate, is also attracting various scientists for its utilization as active and smart packaging materials [44]. Various nano-encapsulated lipids also sought attention for enhanced antioxidant properties of nanocomposites films for enhanced shelf-life of meat products [45,46]. The blend of two or more biopolymer nanoparticles is also able to enhance the physical, mechanical, and thermal properties of various commercially used available polymer packages when incorporated in their matrices [41]. Further examples of preparation techniques of these biopolymer nanoparticles are discussed in the following sections in an in-depth manner within the scope of this paper on meat preservation and processing.

In terms of synthesis, such particles can be prepared using different techniques such as crosslinking, precipitation, emulsification, and coacervation [40]. Covalent or ionic crosslinking methods form polysaccharide-based nanoparticles, by self-assembly and by grafting the hydrophobic part to the backbone of the polymer [47]. For bio-composites formation, starch-based plastics, polyhydroxyalkanoates, cellulose esters, and poly(lactic acid) are the most popular biopolymers [48]. Bio-nanoparticles using chitosan are also increasingly used [49]. Anionic biodegradable polyvinyl alcohol (PVOH) was combined to electrospinning method to form chitosan fibers and ethanolic NaOH solution was used for chitosan fiber stabilization. Further, Okoroh et al. [50] synthesized Zinc Ferrite nanoparticles capped with PVOH, which can be considered an easy, eco-friendly, and cost effective process for thermally treating the biopolymers.

## 3. Main Green Nanoparticle Mechanisms and Sources

Rapid population growth is putting lot of pressure on the global food system, including healthy diets, food safety and security, food supply, and resource sustainability. New technologies based on green nanotechnology are becoming crucial to overcome the challenges related to food security and sustainability.

The extensive usage of synthetic nanomaterials in food packaging fields make them susceptible to being discharged into the atmosphere, various water sources, soil, and landfill waste [51]. In fact, nanomaterials, both organic and inorganic, can be potential pollutants and have remained mostly unidentified due to the limitations of analytical techniques. In the context of promoting sustainability of the environment, much emphasis is currently attributed to green techniques using conditions reacting mildly with nontoxic precursors in the field of nanotechnology [52]. As an alternative, green synthesis of nanoparticles seemed to be a simple, cost-effective, eco-friendly, and relatively reproducible approach [53]. Overall, biological materials provide a greener chemical method to produce materials of very high quality because the biomaterial-based routes eliminate harsh or toxic chemicals. To exemplify this aspect, Table 1 summarizes some of the bacteria, fungi, and plants used for the synthesis of nanoparticles.

**Figure 2:**
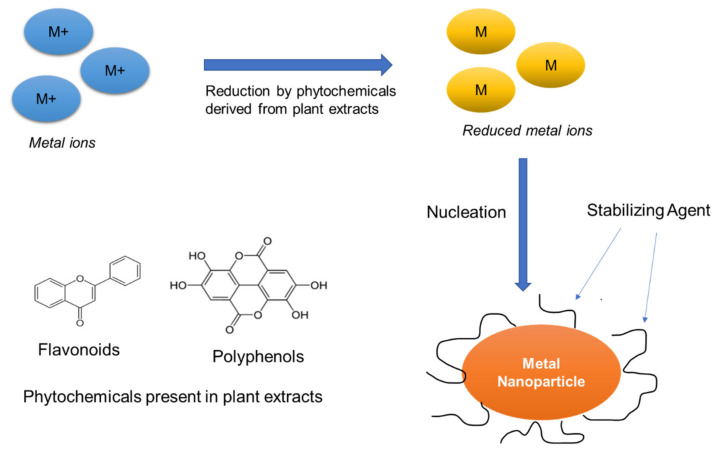
Simplified mechanism of formation of nanoparticles by extracts using plant leaves.

In Figure 2, a summary of nanoparticles formation in the presence of plant extracts is given. The size of nanoparticles can be modulated by varying different parameters such as the quantity of beet juice or ascorbic acid [73,74,75]. Thus, it was seen that bigger silver nanoparticles were formed using lower quantity of beet juice showing an improved catalytic properties and a better stability than those of nanoparticles formed from NaBH_4_ to produce 4-aminophenol from 4-nitrophenol [73]. Bacteria can mitigate metal or heavy metal toxicity using in situ as well as ex situ approaches. Thus, biological reducing agents as well as biochemical paths are used for reducing the metal ions, which are then precipitated to produce the suitable nanoparticles [76]. Enzymes from molds and yeast can be also a valuable source of reducing agents [77]. The sizes of the produced nanoparticles vary with the variation of the solution of metallic ions as well as due to the incubation conditions. However, certain molds act as pathogens for humans thereby limiting their use in nanoparticles synthesis [77]. Biologically synthesized nanoparticles have a functionalized surface containing organic ligands, proteins, polysaccharides, and polyatomic alcohols, contrary to the physical or chemical methods. In this way, size of particles is reduced to enhance their surface area to volume ratio and, therefore, to improve their functional properties such as solubility, absorption, bio-accessibility, and bioavailability, hence facilitating the bioactive agents release [78,79,80].

The use of plant-based ingredients for synthesis of metal nanoparticles is promising as there is no need to use toxic chemicals for the reduction of metal ions and metal oxide. These methods are cost-effective, biocompatible, environmentally friendly, and can be carried out at a large-scale [81]. The synthesis of nanoparticles using leaf extracts of black tea, green tea, eucalyptus leaf, neem leaf enabled the production of gold, silver, copper, zinc, etc., nanoparticles from the extracts [82,83,84]. Algae, fungi, bacteria, and viruses played the role of reducing substances in the synthesis of safe and eco-friendly metallic nanoparticles such as cadmium, gold, platinum, silver, zirconium, palladium, iron, and metal oxides such as titanium oxide (TiO) and zinc oxide (ZnO) [83,85,86].

Recent studies on green-synthesized silver nanoparticles (AgNPs) reported that plant extract(s) or phytochemicals used in the synthesis may contribute to the enhancement of the antimicrobial activities, compared to those synthesized through non-biological routes [87]. For the preparation of nanoparticles in a biogenic way, extracts from amino acids, vitamins, and plants are being widely used for preparing nanoparticles in a less harmful manner [88,89]. It was observed that glucose or fructose that are present in the plant extracts are often responsible for the synthesis of metal nanoparticles. Moreover, a variety of size and shape of nanoparticles could be synthesized from glucose whereas fructose was only capable of making monodisperse silver and gold nanoparticles successfully. Zayed et al. [90] used Fourier Transform Infrared Spectroscopy (FTIR), revealing that the nanoparticles prepared with green synthetic technique from plant extracts are recurrently connected proteins. Plant proteins such as zein, soy protein, and gluten were also identified to be efficient in the synthesis of nanoparticles [91,92]. Likewise, enzymes with a combination of silver by electrostatic forces resulted in the synthesis of nanoparticles owing to their proper structure as well as purity [93]. For instance, for extracellular synthesis of gold nanoparticles, HAuCl_4_ was decreased using a produced α-amylase [94]. Gholami-Shabani et al. [95] used *E. coli* to obtain sulphite reductase enzyme to grow an extract free of any cells for gold nanoparticles synthesis showing antifungal properties and protect humans from the pathogen successfully. *Delftia acidovorans* was found able to synthesize pure gold nanoparticles by inducing proper resistance against that of the gold ions known to be toxic. Viruses are also potent sources to synthesize nanoparticles thanks to the protein present in the outer capsid of the virus providing a very large surface area, that reacts and interacts with metallic ions [96].

Agro-industrial wastes valorization is no longer an option and minimizing the use of toxic solvents and chemicals in nanoparticles synthesis is a must. Accordingly, nanomaterials were also prepared from wastes such eggshells as a part of circular economy, hence reducing the negative environmental impacts [97,98]. In fact, sustainable nanotechnology requires the application of green techniques using mild conditions and no toxic precursors [52]. Even though green nanotechnology showed great progress in several sectors, there is still plenty of room for innovation to expand and create new markets. Nanoparticles were made from wastes and byproducts of seeds and peels of fruit, palm oil, coir of coconut having high amounts of proteins, phenolic compounds, as well as flavonoids that act as reducing agents (for a review: [99]). For example, Ali et al. [100] reported the recovery of fruit waste for the synthesis of silver and gold nanoparticles and their antimicrobial application against food-borne pathogens.

## 4. Applications of Green Nanoparticles in Meat Industry

**Figure 3:**
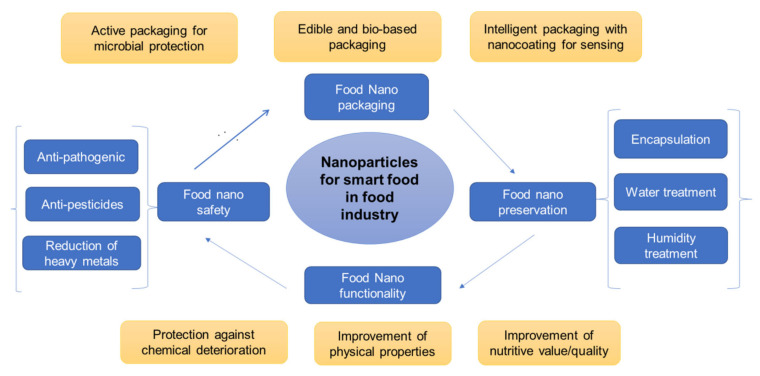
Summary of some applications of nanoparticles for developing smart food in the food industry. Adapted and modified from Bajpai et al., 2018 [102].

The recent advances in the application of nanotechnology in the food industry is fueled by NPs exhibiting superior properties in terms of surface energy, electrical, optical, and mechanical properties than their native counterparts of the same molecular composition. Thus, NPs are being applied in various areas such as the development of smart foods (Figure 3). In food processing, both inorganic and organic NPs (e.g., nanoemulsion and nanofiber) are designed as color/flavor additives, preservatives, or nutraceutical food carriers. In food packaging, inorganic and organic NPs are mainly used as antimicrobial and nanosensors incorporated in films or coating solutions [101]. In the case of meat and meat products, nanotechnology is a rapidly emerging approach to extend the shelf-life acting all along the production chain from processing, preservation to packaging.

### 4.1. Processing

Meat products are often associated with negative health claims due to the high fat and saturated fatty acid contents, and the presence of cholesterol. One of the key challenges facing the meat industry is improving the healthiness of meat products through incorporating health-beneficial ingredients and reducing the use of non-clean label ingredients [103]. To improve the nutritional value of meat products, Weiss et al. [104] suggested the use of plant-based ingredients such as oat fiber, soy fiber, citrus fiber, linseed, flaxseed, and apple pulp as fat replacing agents ensuring the reduction of saturated fatty acids with a possible reduction of salts. In this case, the use of nanoparticles can be a valid approach to reinforce the effect of such replacers thereby improving the antioxidant and antimicrobial delivery of active ingredients [105]. Indeed, Singh et al. [106] proposed that nanoparticles synthesized with green technology could help in the production of meat products in a cost effective way with natural properties by the use of non-chemical ingredients. In terms of processing, the application of nanoparticle paprika successfully enhanced marinating performance and sensory acceptability of marinated meat products [107].

The addition of nanoparticles to processed meat formulations can be a valid strategy of the improvement of functional and nutritional motives. Nanoscale ingredients can be added to meat products to improve taste as well as the texture, while masking off flavors. There is also a potential effect in improving the stability and self-life of meat products, as can be exemplified by the study of Marchetti et al. [108] by the application of bacterial nanocellulose (BNC) to low-lipid low sodium meat emulsions formulated with pre-emulsified high-oleic sunflower oil. It seemed that the addition of a very low concentration of BNC showed a great potential to stabilize meat sausages during 45 days under vacuum storage [108]. In terms of the nutritional modification of pork meat products, ultrasonic-assisted incorporation of nano-encapsulated omega-3 fatty acids was found able to enhance the fatty acid profile (e.g., n3/n6 ratio values) of the generated product [19].

The meat synthetic preservatives such as nitrites can prevent undesirable changes in meat products but have adverse effects on consumer’s health. Natural preservatives, especially nanoscale plant-origin materials such as nanoemulsions, can be helpful to solve this problem. In this context, the combinations of essential oils (EOs) and their nanoemulsions acted as an antioxidant without affecting the technological characteristics of the meat product [20]. For example, Noori et al. [109] applied a sodium caseinate containing a nanoemulsion of ginger EO on chicken breast fillets, achieving a significant decrease in the total aerobic psychrophilic bacteria of the refrigerated chicken fillets. Another study compared *Trachyspermum ammi* EO in both forms, emulsion and nanoemulsions, in alginate-based edible coatings against inoculated *Listeria monocytogenes* in turkey fillets for a period of 12 days [110]. The results showed that the highest anti-*Listeria* activity was observed in the case of nanoemulsion coating instead of the emulsion practice. Hasani-Javanmardi et al. [111] investigated the effects of safflower oil nanoemulsion and cumin EO combined with O_2_ absorber packaging on the quality and shelf-life of refrigerated lamb loins. Nanoemulsions were found appropriate systems to encapsulate hydrophobic compounds and effectively introduce them into meat active packaging. Stable thyme EO chitosan nanoemulsions and thymol chitosan nanoemulsions were recently developed [112]. These nanoemulsions acted as natural novel antibacterial packaging materials resulting in an effective meat preservation. UV–Vis light barrier property is one of the key features in the development of films for specific food packaging because of it is ability to avoid or retard the peroxidation of lipids, pigments, proteins, or vitamins. This feature is directly related to the food shelf-life by preventing undesirable flavors, color, odors, loss of nutrients, thus preserving organoleptic and nutritional properties of the packed food. Similar to metal nanoparticles, metal oxide nanoparticles have gained great attention as antimicrobial agents in food packaging because of their ability to absorb UV and photocatalytic disinfecting character. For instance, TiO_2_ nanoparticles have been observed to be effective against common foodborne pathogens and viruses under UV illumination but not in the dark [113]. In principle, meat packaging films incorporating TiO_2_ nanoparticles may have the additional benefit of protecting foods from the oxidizing effects of UV irradiation [114].

The detection of contaminants in the meat industry is crucial for safety and security measures. Indeed, contaminants can cause detrimental effects to consumers, such as allergic reactions, carcinogenic or teratogenic mechanisms, or induce antimicrobial resistance. Contaminant sensors can be used to detect banned adulterants, pharmacological residues such as antibiotics and hormones, and allergens [115,116]. To detect antibiotics even at an extremely low level, gold nanoparticles-based biosensors were used to capture antibiotics [117]. Furthermore, the presence of β-agonists in meats has received a special attention around the world due to its potential threat to public health [118,119]. To achieve a rapid and onsite detection of β-agonists, many nanoparticle-based sensors have been developed as a promising complementary analytical tool [120,121].

Unlike fresh meat, processed meat products contain sodium/potassium nitrite and nitrate, which must be declared on the label among the potential list of allergens [122]. Therefore, the use of nanotechnology for rapid, simple, and accurate monitoring of nitrite/nitrate is highly desirable [123].

### 4.2. Preservation

Many techniques for preserving meats have evolved over time, from salt addition to drying, refrigeration, and nanotechnology [124,125,126]. Recently, Alirezalu et al. [127] used ε-polylysine along with ε-polylysine nanoparticles from plant extracts (olive and green tea) in formulating nitrile-free sausages. The use of nanoparticles extended the shelf-life and considerably improved the microbiological safety of sausages. Indeed, silver ions are known to be slowly released from AgNPs and act as antimicrobial agents against a broad spectrum of Gram-negative and Gram-positive bacteria, fungi, protozoa, and certain viruses [128]. More specifically, cellulose pads containing AgNPs generated from silver ions in situ have been shown to reduce the microbial levels of exudates from beef meat stored in modified atmosphere packaging [129]. Silver nanoparticles made from tea leaves extract were proven to have an antimicrobial activity [130]. This can be used in packaging of meat products against microbial damage such as Gram-negative pathogens. Ghaderi-Ghahfarokhi et al. [131] reported that beef burgers enriched with thyme phenolics-loaded chitosan nanoparticles resulted in positive changes in antioxidant capacity, overall acceptability, and sensory quality of beef burgers during refrigerated storage.

**Figure 4:**
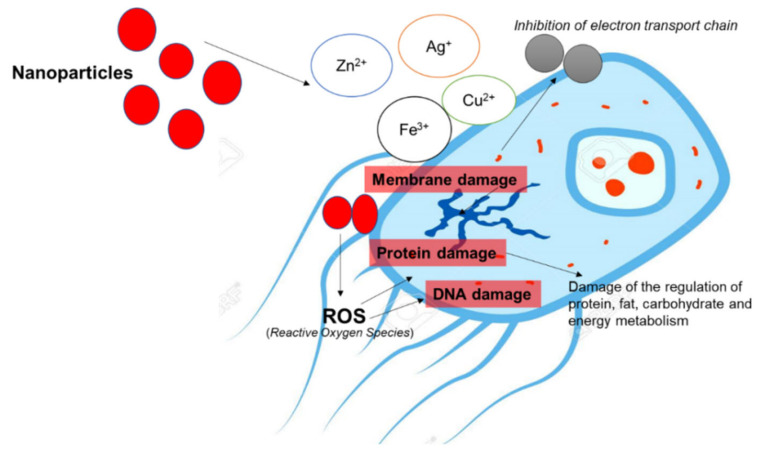
Simplified mechanism of nanoparticle action in the bacterial cells.

Sunflower oil-based nanoemulsion used in steaks made from Indo-Pacific king mackerel resulted in reducing the growth of microbes, thereby increasing the shelf-life [132]. Moreover, nanoparticles exhibit an attractive antibacterial activity due to their increased specific surface area leading to enhanced surface reactivity [133]. Figure 4 illustrates nanoparticles’ mechanism in damaging the membrane, bacterial protein, and bacterial DNA. The possible mechanism of antibacterial behavior of nanoparticles relies on the interaction of nanoparticles with bacteria, (i) excessive ROS generation and (ii) precipitation of nanoparticles on the bacterial exterior; which disrupts the cellular activities, resulting in membrane disturbance [133].

Nanocapsules charged with tarragon EOs can be combined with chitosan–gelatin-based films to significantly inhibit the deterioration of pork slice quality, as nanoencapsulation contributes to the sustained release of tarragon EOs, resulting in improved antioxidant, antibacterial, and sensory properties [134]. As a new approach, Esmaeili et al. [135] have successfully incorporated nanoencapsulated garlic essential oil into edible films for extending shelf-life of vacuum-packed sausages. Similar findings were reported by Pabast et al. [36], who compared the effect of chitosan-containing, -free, or -nanoencapsulated *Satureja khuzestanica* essential oils on the quality of lamb meat. The results showed that nanoencapsulated EOs could improve microbiological safety and extend shelf-life during chilled storage of lamb meat. Recently, Bahrami Feridoni et al. [136] demonstrated that nanoencapsulation of sour tea (*Hibiscus sabdariffa* L.) extracted with carboxymethylcellulose extended significantly the shelf-life of chicken nuggets, therefore reducing the oxidative effect on the final meat product. In agreement, carboxymethyl cellulose-based functional films incorporated with zinc oxide nanoparticles provided an excellent antioxidant activity and 100% UV protection in high-fat meat products [16].

### 4.3. Packaging

Industrial applications of nanotechnology quickly introduced improvement in packaging materials of meat product to ensure safety, nutritional and organoleptic qualities, and their continuous monitoring during product shelf-life. The invention of active and smart packaging, nanosensors, greatly contributed in maintaining and improving quality and safety. For example, Gedarawatte et al. [137] explored the potential of bacterial nanocellulose loaded with nisin against selected meat spoilage such as lactic acid bacteria and concluded that nisin-loaded bacterial nanocellulose may be used as antimicrobial agents in active food packaging.

Selenium (Se) nanoparticles were found more stable because of the phytochemicals present in plant extracts acting as natural stabilizers [138]. Simultaneous incorporation of okra mucilage and ZnO nanoparticles into a carboxymethylcellulose-based film extended the shelf-life of chicken meat [139]. It was also reported that the use of nanoparticles can be beneficial in providing augmented mechanical and heat resistance to the packaging of the meat products [140]. Indeed, nanocomposites can be used to modify the plastic as an excellent barrier in a similar way to that of metal or glass packaging. Biopolymer or green coatings polymers added with nanoparticles could also act as promising materials for renewable packaging and therefore act as alternatives for polymers based on petroleum.

Edible films made with silver nanoparticle coating of turkey meat were found efficient against pathogenic microorganisms [141]. Moreover, polylactic acid films with cinnamon essential oil and silver–copper nanoparticles reduced bacterial spoilage of packaged chicken meat compared to fresh meat product [142]. Similarly, polylactic acid film incorporated with nanochitosan and *Polylophium involucratum* essential oil prolonged the shelf-life of chicken fillet during storage (up to 10 days) without any adverse sensorial properties [143]. In agreement, nano-size curcumin and rosemary oil limited the microbial spoilage of rainbow fillets [144]. A novel active packaging material based on nanocomposite-chitosan and nanocellulose incorporated with different concentrations of *Ziziphora clinopodioides* EO alone and in combination with *Ficus carica* extract was investigated in a minced camel’s meat to increase the shelf-life and inhibit the growth of *Listeria monocytogenes* and *Escherichia coli* O157:H7 during storage at refrigerated condition [145]. The results indicated that nanomaterials could be considered as promising packaging materials for minced camel’s meat.

Direct use of nanomaterials during animal farming, food processing, and product storage levels can lead to the presence of such materials in the final product. In this context, analytical methods for the detection and characterization of nanomaterials in complex food matrices and toxicological data are strongly needed to assess the risk for consumers. A different approach for the quantification and characterization of silver nanoparticles in chicken meat consists of inductively coupled plasma mass spectrometry (ICP-MS), which has been used either alone [146], or in combination with asymmetric flow field-flow fractionation (AF-FFF) [147,148]. Cushen et al. [149] attempted to measure the migration of AgNPs from poly vinyl chloride (PVC) matrix into chicken meat by using an ICP-MS method. The obtained results showed that the measured amount of AgNPs did not exceed the limits set by the European Union regulations.

In their study, Khalaf et al. [150] have proven the stability and antimicrobial effect of pullulan edible films incorporated with AgNPs nanoparticles on turkey deli meat quality. Other earlier studies further reported the effective microbial preservation effect of AgNPs absorbent pads against *Escherichia coli* and *Staphylococcus aureus* poultry meat [151]. In the same context, the group of Martinez-Abad and co-authors have developed ethylene-vinyl alcohol films with AgNPs with a great effect against *Listeria monocytogenes* in chicken and pork meats [152]. Further, the antimicrobial capacity of low-density polyethylene blended with Ag and ZnO nanoparticles against *E. coli*, *Pseudomonas aeruginosa*, and *Listeria monocytogenes* on chicken breast meat was also evidenced [153]. PLA-Chitosan coating solutions and films applied to ready-to-eat deli meat exhibited an efficient antimicrobial efficacy against *Listeria innocua* [154]. Further studies reported interesting findings using portable chitosan-ZnO nanocomposite pouches with antibacterial activity against *E. coli* and *Staphylococcus aureus* of raw meat [155] or of glycyrrhiza polysaccharide loaded with tea tree EOs encapsulated into gliadin nanofibers stabilized with arabic gum to inhibit the growth of *Salmonella typhimurium* on the surface of meat products [156].

An interesting study on chicken active packaging was conducted by Ahmed et al. [157] where the authors prepared compression molded poly-lactic active films loaded with bimetallic copper and silver nanoparticles capped with cinnamon essential oils. The composite films exhibited enhanced moisture barrier properties and served as excellent antimicrobial inhibitory agents against pathogenic bacteria likely *Salmonella typhimurium, Campylobacter jejuni*, and *Listeria monocytogenes* during 21 days of storage. Azarifar et al. [158] studied the shelf-life characteristics of beef wrapped in gelatin carboxymethyl cellulose films incorporated with chitosan nanofibers and *Trachyspermum ammi* essential oil. The film containing 2 and 4% of nanocomposite and 0.64% of essential oil inhibited the psychotropic bacterial growth for almost 15 days. In addition, the films incorporated with 1% essential oil retarded significantly the lactic acid bacteria growth. More interestingly, the essential oil reinforced nanocomposite wraps were extensively effective to delay lipid oxidation, protein degradation thereby enhancing positively the shelf-life and sensory qualities of beef cuts. In another work by Pires et al. [159], solution casted bionanocomposite films of chitosan/montmorrilonte incorporated with rosemary–ginger and essential oil were prepared for utilization as a preservative to pack chicken fillets and shelf-life trial. The composite films enabled extending the shelf-life by reducing half of the microbial count during 15 days storage and delaying lipid oxidation in fresh poultry meat.

### 4.4. Toxicity Issues

The great development of nanotechnology in recent years and the increase in global food trade have highlighted the importance of developing reliable safety assessment measures and establishing strict guidelines to ensure proper functioning and consumer safety. Although nanotechnology may hold great promise to improve the quality and microbial safety of meats, there are concerns related to the uncertainty of toxicological effects, health risks, and environmental impact [160]. The increasing number of publications and patents highlights the fast growth of this topic in the agro-food industry, which is confirmed by the significant number of companies using nanotechnology in the development of their products. However, the acceptance of this novel technology in various aspects of food preservation and safety requires further studies to establish absence of adverse effects of nanomaterials and their safe use on foods and food contact surfaces.

Accordingly, Table 2 summarizes in a non-exhaustive manner some of the studies that investigated the toxicity of nanoparticles through in vitro and in vivo studies using different cells and for more detail we invite the reader to specialized reviews and meta-analysis in this field [161,162]. Further, the direct use of nanomaterials during animal farming, food processing, and product storage levels can lead to their presence in the final product. In this context, advanced analytical methods are required for the detection, characterization, and risk assessment of nanomaterials, including in meat products and used nano-packages, since the literature is scarce.

Despite the high potency in enhancing shelf-life and safety of food products, the issue of nanoparticle induced packaging material is a major concern among researchers. Reports state that fast oxidation in chicken sausages coated with silver nanoparticles was observed after a storage of 15 days [172]. The concentration present in sausages was reported to be less due to processing and cooking steps whereas texture analysis parameters like chewiness, gumminess, and cohesiveness, were drastically impacted after 15 days of storage, which may be due to the interaction of silver nanoparticles and meat proteins, in support to a previous study [173]. It is worth noting that silver was detected in body tissues such as the glomeruli, the skin epidermis, and the intestines after exposure to both ionic and nanoparticulated silver suspensions (Table 2, and for a comprehensive review refer to [161]).

Silver and copper nanoparticles are being introduced in polymer matrices for active packaging of meat products due to the promising antibacterial effects. Their safety issue is also raising awareness due to human exposure (Table 2), more specifically during food consumption. Thus, researchers are more focusing on migration studies of these metal nanoparticles in food with, for instance, the help of mathematical modeling [149]. The reports are nearly correct to the laboratory assessment value in case of quantification of silver nanoparticles (0.003–0.005 mg/dm^2^) released from polymer matrix but less accurate for the quantification of copper nanoparticles (0.024–0.049 mg/dm^2^). It seemed then that maintaining safety standards according to European legislation, silver and copper nanoparticles can be recommended as potential safe antimicrobial agents for active packaging as the migration value of these nanoparticles is less than the actual amount of dietary intake level. The food additive E174 that is AgNPs is nowadays recognized as a relevant route of human exposure to silver with an average exposure of around 30% of total dietary exposure to silver [174]. However, it is important to note that the amount of silver ions released from AgNPs used as a food additive or in the packages of meat products remains uncertain due to the multiple factors simultaneously driving the release such as the pH of the medium, which includes the NPs capping agent, or the particle size, among others [162,174]. These considerations make evident the relevance of determining nanoparticles’ fate, including AgNPs, after human ingestion for potential adverse health effects and risk assessment evaluation. Ramos et al. [173] revealed in their simultaneous characterization of AgNPs and in vitro human gastrointestinal digestion of dissolved silver in chicken meat using single particle inductively coupled plasma mass spectrometry, that only 13% of the AgNPs present in the reference material would reach the intestine wall in the case of spiked chicken meat. Meanwhile, other bioaccessible dissolved forms of silver would account for as much as 44% of the silver initially spiked to the meat paste. The findings of this study evidenced successive transformation of the AgNPs size and dispersion stage during the salivary, gastric, and intestinal digestions as a result of the temperature and pH shifts and variable concentrations of enzymes and salts. The nature and extension of the transformations seemed to be related to the presence of other food components, especially of proteins.

A commercially available food packaging improved with AgNPs, intended to package chicken meat, was evaluated to determine silver migration into packaged food, under common domestic storage conditions [175]. The migration detected was reassuringly slow. Recently, Liu et al. [176] reported that there is no significant toxicity relationship between nanomaterials and their use in the food industry. According to Otles et al. [177], a bioassay based on nanoparticles was prepared for the purpose of rapid detection of a dangerous food borne disease in food caused by *E. coli* O157:H7. Silica nanoparticles having 60 nm diameter along with antibodies and dye molecules reacting with the surface antigens of the bacteria were used for the detection. When the antigen of the bacteria and antibodies in the nanosensors react, a fluorescent signal is emitted which can be detected with the help of spectro-fluorometric analysis successfully. Fuertes et al. [178] highlighted the interest of applying nanotechnology in packaging to provide an accurate monitoring, tracking, as well as communication during the whole production chain.

In support of these examples, the study by Kittler et al. [179] observed that AgNPs dissolve slowly into ions in a time scale of several days (a trial of 125 days at 5, 25, and 37 °C). From this study, it seemed that the biological action of freshly prepared and aged nanoparticles is strongly different due to the different amount of released ions. Thus, the level of the toxicity would linearly depend on the level of exposure and the association with other nanoparticles. Taken all together, it is today a burden to define and use appropriate reference food materials and realistic physiological conditions for appropriate risk assessment evaluation associated to AgNPs oral ingestion as well that of other nanoparticles. In this context, the comprehensive review of McCracken et al. [162] stated that although no estimates of human consumption of AgNPs and zinc oxide (ZnO) as well as other NPs are currently available, considering that food-related ZnO and AgNP use is for the most part limited to food packaging and the amount of NPs leaching from the packaging into the food product is suggested to be low, levels of gastrointestinal tract exposure are likely to be lower than for silica and titanium dioxide (TiO_2_). However, dosage in in vitro and in vivo experiments conducted with Ag and ZnO NPs has generally found to be have been of similar magnitude as those conducted with silica and TiO2 (the reader can refer to the supplementary data of McCracken et al. [162] that analyzed toxicity of nanoparticles of all the studies published since 2012). Thus, more research designs should be implemented to carry out food surface mitigation studies or release kinetics mechanism of nanoparticles onto food surfaces.

Nanosensors are known as effective tools to detect the presence of pathogens, molecules, or gases (Table 3). For instance, electronic tongues having nanosensors in food packaging detected the release of gases from spoiled food and indicated the freshness level of the food [180]. Similarly, silver nanoparticles enhanced antimicrobial activity against *Bacillus cereus* and *Escherichia coli* when implemented in combination with titanium dioxide (TiO_2_) as well as carbon nanotubes [181]. Thus, packaging using nanoparticles can protect against toxicity and spoilage for fresh and processed meat products [181,182].

The lack of standardized or validated nanotoxicity evaluation methods resulted in data inconsistency among published studies, and thus limited the development of robust strategies for nanoparticles risk assessment. More specifically, there are concerns about the fact that nanoparticles might cross biological barriers and, due to the increase surface-to-mass ratio and surface reactivity, new potential toxicological properties can occur.

## 5. Future Paradigm of Meat Industry

Meat is generally considered to be an active (dynamic) system having a very limited shelf-life period and is prone to alterations in the sensory features throughout the storage period owing to alterations in chemical, physical, or microbiological environment [198]. Implementing nanotechnology in meat processing and packaging has the aims to bring antimicrobial and barrier properties along with improving sensory characters or/and encapsulating the bioactive compounds with nanoparticles [131,199]. Analytical nanometrology is challenging and it is necessary to recognize that the detection and determination of nanocomponents in complex meat products is still very limited, and no systematic validated standard is currently available yet.

Plant extracts and EOs have good preservative properties, but their strong flavors and adverse effects on sensorial attributes might limit their utilization in meats. Encapsulation could be a useful strategy to overcome negative sensory attributes of the final products. Nanocolloidal substances were reported interesting to be used in packaging and processing to obtain safe and healthy foods [200]. Nevertheless, further investigations are required to understand the behavior and features of the nanocolloids. In summary, the current research on nanotechnology application in meat industry is focused on applying the polymer-based nanomaterials to improve packaging, smart packaging, as well as active packaging [201]. Considering that, certain parameters such as pH, temperature, or gas level changes can affect the quality, safety, or freshness of packaged products. In the case of improved packaging, nanocomposites can be of great importance because they will reinforce the polymer matrix thanks to nanoscale fillers like cellulose whiskers, clay, and silicates, thereby maintaining product quality and avoiding spoilage [202]. Hadian et al. [203] reported that nano-encapsulated *Rosmarinus officinalis* EOs in chitosan-benzoic acid nanogel improved the shelf-life of beef steaks because nanogels reduced *Salmonella Typhimurium* during refrigerated storage. Furthermore, nanoparticles from lyophilized pomegranate peel prevented lipid oxidation and improved microbial quality and cooking characteristics of minced beef [199]. Similarly, Ghaderi-Ghahfarokhi et al. [131] noticed that thyme EOs encapsulation in chitosan nanoparticles is a promising strategy to control the undesirable lipid oxidation and sensory changes in beef burgers.

The pork gelatin, which is the most common form of gelatin, is not acceptable to some consumers because of religious and dietary preferences. Natural camel skin gelatin nanoparticles revealed high suitability for food applications, with a potential use in the growing global halal food market [204]. The implementation of nanocomposites in the food industry gave rise to issues causing an impact on the environment, as they are not degradable in nature. There are still limited studies on the eco-toxicity of the nanoparticles, which must be further carried out in abundance to ensure safety [205,206]. The use of nanotechnology has not been thoroughly studied so far, in the context of its toxicity. Mathematical models or response surface methodology can be utilized in a smart way to measure the toxicity of the nanoparticles used in a food system.

## 6. Conclusions and Future Prospects

Nanotechnology can play different roles in the food chain, especially that of the meat production industry from processing, preservation to packaging of fresh or processed meat products. It is highly important to choose very carefully the nanomaterials that would ensure both safety and final desired quality of the products. Moreover, packaging materials should be made of biodegradable biopolymer for sustainability concerns. Zinc oxide and magnesium oxide nanoparticles have been reported to be suitable as packaging materials along with amorphous silica nanoparticles. The advancements in nanotechnology can supposedly bring an all-new digital future, which can be regarded as big data information, thereby leading to new research fields. Moreover, the future perspective of nanotechnology is increasingly focused on using carbon nanotubes as materials for packing meat products to readily detect spoilage organisms and toxic proteins.

Nanosensors are valuable weapons to detect any kinds of pathogens, even at low amounts. Consumers are showing more acceptance of nanomaterials as materials in packaging but to less extent as ingredients in meat products. This is due to unfamiliarity with nanoparticles and the lack of knowledge about their composition and role in foods. Only after determination of the safety of the nanomaterials in food processing and the packaging aspect, it is possible that the public will accept this technology. It is highly essential to educate the public about the advantages, safety aspect, health aspect, as well as environmental impact related to implementation of nanotechnology in the food industry.

The exclusive and innovative properties of the nanomaterials will urge the food industry to increase commercial applications of the technology. Robust studies on toxicity issues related to the nanomaterials are crucial to ensure safety and security. Standard regulation is indeed required to provide guidelines for assessing the risks. Future trends of nanotechnology will address the challenge of developing healthier, safer, and more sustainable nanoparticles.

## Figures and Tables

**Table 1:** A non-exhaustive list of nanoparticles from different bio-sources (bacteria, fungi, and plants) prepared by green technology with application in food/meat products.

Bio-Sources	Nanoparticles	Size Range of the Nanoparticles	Morphological Feature of the Nanoparticles	Applications and Effects	References
*Lactobacillus casei* (Bacteria)	Silver (Ag) nanoparticles	20–50 nm	Spherical	Bio-labeling in food products	[54]
*Bacillus cereus* (Bacteria)	Silver nanoparticles	20–40 nm	Spherical	Food packaging as it exhibits antibacterial protection against harmful food pathogens like *Staphylococcus aureus, Pseudomonas*, and *Escherichia coli*	[55]
*Rhizopus nigricans*	Silver nanoparticles	35–40 nm	Round	Has bactericidal effect which can be used in food packaging	[56]
*Aspergillus terreus*	Zinc oxide nanoparticles	8 nm	Spherical	Highly applicable in case of biosensing in food packaging	[57]
*Acalypha indica* (Plant)	Silver nanoparticles	20–30 nm	Spherical	Exhibits antibacterial protection against various types of pathogens and thus can be used in food packaging	[58]
*Camellia sinensis* (Plant)	Gold and silver nanoparticles	20 nm	Spherical and prismatic	Act as excellent sensors agents with potential use in food packaging	[59]
Unripe Papaya (Fruit)	Silver nanoparticles	<100 nm	Spherical	Beef preservation	[60]
*Eucalyptus camaldulensis* leaf extract	Silver nanoparticles	138.6 nm	Spheroid	Active food packaging	[61]
*Nigela sativa* seed extract	Zinc oxide nanoparticles	~24 nm	Hexagonal wurtzite structure	Active food packaging	[62]
Avocado seed extracts	Silver nanoparticles	15–25 nm	Spheroid clusters	Food packaging with protective action against pathogens	[63]
*Lysiloma acapulcensi* (medicinal plant)	Silver nanoparticles	5 nm	Spherical and quasi-spherical	Food packaging with protective action against pathogens	[64]
Dry baker’s yeast	Silver nanoparticles	13.8 nm	Spherical	Antibacterial activity against *E. coli*	[65]
*Aspergillus flavus* NJP08	Silver nanoparticles	17 ± 5.9 nm	Spherical	Stability of product	[66]
*Tabernaemontana heyneana Wall* (leaf, stem, callus extract)	Zinc oxide nanoparticles	50–100 nm	Clustered	Antioxidant activity	[67]
*Ruta graveolens*	Zinc oxide nanoparticles	20–30 nm	Hexagonal, spherical agglomerate	Antibacterial activity against enterogenic pathogens and antioxidant activity against free radicals	[68]
*Trigonella foenum-graecum* (Fenugreek) seed extract	Silver nanoparticles	82.53 nm	Spherical	Biocidal potency against both Gram positive and Gram negative bacteria	[69]
*Trichoderma harzianum*	Silver, zinc oxide, and copper oxide nanoparticles	5–18 nm (silver), 38–77 nm (copper) 134–200 nm	Spherical (silver), elongated fibers (copper oxide nanoparticles), fan and bouquet structure (zinc oxide nanoparticles)	Inhibitory action against *A. alternata, P. oryzae*, and *S. sclerotiorum*	[70]
*Stereum hirsutum*	Copper and copper oxide nanoparticles	5–20 nm	Spherical	Antibacterial activity	[71]
Black current and apricot pomace waste extracts	Silver nanoparticles	40–60 nm	Spherical	May be used as food packaging as it exhibits an antibacterial effect against Gram negative bacteria	[72]

**Table 2:** A non-exhaustive list of some examples of toxicity of nanoparticles gathered from in vitro and in vivo studies.

Nanoparticles	Particle Size	Administration Mode	Species/Cell Culture	Effects	References
Silver	20 and 40 nm	In vitro	Human leukemia cell	Cell viability decreased with increased concentration of NPs	[163]
Silver	30 and 50 nm	In vitro	Human alveolar cell line	Cell viability decreased with increased concentration of NPs	[164]
Iron oxide NPs	30 nm	In vitro	Murine macrophage cells	Cell viability decreased	[165]
Fullerenes	178 nm	In vitro	Chinese hamster ovary cells; Human epidermoid-like-carcinoma cells; Human embryonic kidney cells;	DNA strand breakage chromosomal damage	[165]
Copper	80 nm	Oral ingestion	Rats	—Liver injury —Oxidative stress —Inflammatory reactions	[166]
Gold	4, 10, 28 and 58 nm	Oral ingestion	Mice	Gold nanoparticles were captured by the gastrointestinal tract and translocated by blood to other organs such as liver, spleen, kidney, heart, lungs, spleen, and brain	[167]
Polystyrene microspheres	50, 100, 300 nm	Oral gavage	Rats	Accumulation in liver and spleen via lymph	[168]
Titanium oxide	160 nm	Oral ingestion	Mice	DNA damage and genotoxicity	[165]
Chitosan nanoparticle	200 nm	In vivo	Zebrafish	Malformations including a bent spine, pericardial edema, and an opaque yolk in zebrafish embryos; increase of heat-shock proteins	[169]
Colloidal silica	20 and 100 nm	Oral ingestion	Mice	Increase of white blood cells Differential expression of cytokines	[170]
Zinc oxide	20 nm	Oral gavage	Rats	Microscopic lesions in liver, pancreas, heart, and stomach	[171]

**Table 3:** A non-exhaustive list of some nanobiosensors used in the field of food/meat technology.

Nanomaterial-Based Biosensors	Mode of Action/Technology	Application and Main Objectives	References
Chitosan	Agents for coating of food products	Senses any kind of fungal development	[183]
Carbon nanotubes	Electrical, thermal, mechanical, and optical conductivity	Inspection of food as well as vacuum proof packaging of food	[184]
Gold nanoparticles	Gold nanoparticles integrated with either DNA, enzymes, or antibodies	Used in meat industry for shelf-life enhancement	[185]
Graphene	Nanocomposites based on nanoplates	Detection of any sort of contaminants in the food products	[186]
Allyl isothiocyanate	Antimicrobial packaging substance	Better and effective storage of shredded and cooked chicken meat	[187]
Gold nanoparticles	Gold nanoparticles combined with swine specific oligo probe	Detection and quantification of pork adulteration	[188]
Gold nanoparticles	Biogenic amines composite prepared	Poultry spoilage detection	[189]
Silica nanoparticle	Silica nanoparticle enhanced Dot Blot DNA biosensor	High sensitivity detection of *Campylobacter* sp. in chicken	[190]
Citrate-tannate coated gold nanocrystals	Nanocrystals attenuated with gene probe	Detection and quantification of DNA sequences in degraded mixed meat	[191]
Nano-biosensor from immunomagnetic beads and quantum dots	Detection of enrofloxacin	Rapid and cost-effective test to detect chicken spoilage	[192]
Graphene/Titanium oxide nanocomposite	Electrochemistry of xanthine oxidase, anti-interference properties in presence of uric acid, ascorbic acid, and glucose	Freshness detection in pork	[193]
Carbon black blended with non-conductive polymers	Polymerase chain reaction	Detection of volatile compounds released and foodborne pathogenic contamination in beef and sausages	[194]
AgNPs/Polyetherimide composite	Ion-exchange mechanism	Freshness detection in turkey, chicken, and salmon	[195]
Molecularly imprinted nanogels	Molecular imprinted technology	Porcine serum albumin detection in raw meat extract for halal control	[196]
Bio-composite of multi-walled carbon nanotube and poly(l-aspartic acid)	Enzyme immobilization	Meat freshness indication	[197]

## Data Availability

Not applicable.
