# Correction: Lamri et al. Nanotechnology as a Processing and Packaging Tool to Improve Meat Quality and Safety. *Foods* 2021, *10*, 2633

**DOI:** 10.3390/foods15030513

**Published:** 2026-02-02

**Authors:** Melisa Lamri, Tanima Bhattacharya, Fatma Boukid, Imene Chentir, Amira Leila Dib, Debashrita Das, Djamel Djenane, Mohammed Gagaoua

**Affiliations:** 1 Laboratory of Food Quality and Food Safety, Department of Food Technology, Université Mouloud Mammeri, Tizi-Ouzou 15000, Algeria; lamrimeliza1@gmail.com (M.L.); djenane6@yahoo.es (D.D.); 2 Innovation, Incubation & Industry (I-Cube) Laboratory, Techno India NJR Institute of Technology, Udaipur 313003, India; btanima1987@gmail.com; 3 Food Safety and Functionality Programme, Institute of Agriculture and Food Research and Technology (IRTA), 17121 Monells, Spain; fatma.boukid@irta.cat; 4 Laboratory of Food, Processing, Control and Agroressources Valorization, Higher School of Food Science and Agri-Food Industry, Algiers 16200, Algeria; i.chentir@essaia.dz; 5 GSPA Research Laboratory, Institut des Sciences Vétérinaires, Université Frères Mentouri, Constantine 1, Constantine 25000, Algeria; dibamira@hotmail.com; 6 School of Community Science & Technology, IIEST Shibpur, Howrah 711103, India; debashritadas95@gmail.com; 7 Food Quality and Sensory Science Department, Teagasc Food Research Centre, Ashtown, D15 KN3K Dublin, Ireland

In the original publication [1], citation [102] was missing and inadvertently not cited due to an error in forgetting to acknowledge the modification and use of the figure from Bajpai et al. during the preparation of the article. The citation has now been inserted at the end of Figure 3’s caption and should read:

**Figure 3.** Summary of some applications of nanoparticles for developing smart food in the food industry. Adapted and modified from Bajpai et al., 2018 [102].

102. Bajpai, V.K.; Kamle, M.; Shukla, S.; Mahato, D.K.; Chandra, P.; Hwang, S.K.; Kumar, P.; Huh, Y.S.; Han, Y.K. Prospects of using nanotechnology for food preservation, safety, and security. *J. Food Drug Anal.* **2018**, *26*, 1201–1214.

With this correction, the order of some references has been adjusted accordingly. The authors state that the scientific conclusions are unaffected. This correction was approved by the Academic Editor. The original publication has also been updated.
